# The promise of multi-omics approaches to discover biological alterations with clinical relevance in Alzheimer’s disease

**DOI:** 10.3389/fnagi.2022.1065904

**Published:** 2022-12-07

**Authors:** Christopher Clark, Miriam Rabl, Loïc Dayon, Julius Popp

**Affiliations:** ^1^Department of Psychiatry, Psychotherapy and Psychosomatics, University of Zürich, Zürich, Switzerland; ^2^Geriatric Psychiatry, University Hospital of Psychiatry Zürich, Zürich, Switzerland; ^3^University of Lausanne, Lausanne, Switzerland; ^4^Nestlé Institute of Food Safety and Analytical Sciences, Nestlé Research, Lausanne, Switzerland; ^5^Institut des Sciences et Ingénierie Chimiques, Ecole Polytechnique Fédérale de Lausanne, Lausanne, Switzerland; ^6^Old Age Psychiatry, Department of Psychiatry, Lausanne University Hospital, Lausanne, Switzerland

**Keywords:** multi-omics, Alzheimer’s disease, integration, clinical relevance, biomarkers, personalised medicine

## Abstract

Beyond the core features of Alzheimer’s disease (AD) pathology, i.e. amyloid pathology, tau-related neurodegeneration and microglia response, multiple other molecular alterations and pathway dysregulations have been observed in AD. Their inter-individual variations, complex interactions and relevance for clinical manifestation and disease progression remain poorly understood, however. Heterogeneity at both pathophysiological and clinical levels complicates diagnosis, prognosis, treatment and drug design and testing. High-throughput “omics” comprise unbiased and untargeted data-driven methods which allow the exploration of a wide spectrum of disease-related changes at different endophenotype levels without focussing *a priori* on specific molecular pathways or molecules. Crucially, new methodological and statistical advances now allow for the integrative analysis of data resulting from multiple and different omics methods. These multi-omics approaches offer the unique advantage of providing a more comprehensive characterisation of the AD endophenotype and to capture molecular signatures and interactions spanning various biological levels. These new insights can then help decipher disease mechanisms more deeply. In this review, we describe the different multi-omics tools and approaches currently available and how they have been applied in AD research so far. We discuss how multi-omics can be used to explore molecular alterations related to core features of the AD pathologies and how they interact with comorbid pathological alterations. We further discuss whether the identified pathophysiological changes are relevant for the clinical manifestation of AD, in terms of both cognitive impairment and neuropsychiatric symptoms, and for clinical disease progression over time. Finally, we address the opportunities for multi-omics approaches to help discover novel biomarkers for diagnosis and monitoring of relevant pathophysiological processes, along with personalised intervention strategies in AD.

## Introduction

The hallmarks of Alzheimer’s disease (AD) are amyloid aggregation, tauopathy and neuronal injury. Along with the microglia response, these are considered as the “core” AD pathology (Scheltens et al., 2021). Indeed, cerebrospinal fluid (CSF) levels of Aβ1-42, total-tau and tau phosphorylated at threonine 181 (p-tau181) levels, mirroring cerebral amyloid, neuronal injury and tau pathology, are currently the best validated biofluid markers of AD pathology. However, these pathology aspects do not fully explain the vast heterogeneity of the observed disease phenotypes. A large body of evidence indicates that other biological pathways and molecular alterations happening at both cerebral and systemic levels are involved in the pathophysiological processes underlying AD. These processes may substantially contribute to the development and acceleration of amyloid pathology and neurodegeneration, precipitate the manifestation of cognitive and neuropsychiatric symptoms (NPS) and may therefore represent targets for both interfering with the developing AD pathology and slowing down or even reverse clinical disease progression. For example, neuroinflammation was proposed to play a key role at the interface between both tau and amyloid pathologies (Calsolaro and Edison, 2016; Luca et al., 2018; Marttinen et al., 2018). Alterations of the lipid metabolism including oxysterols (Gamba et al., 2019; Jahn et al., 2021), cholesterol and non-cholesterol sterols (Popp et al., 2012, 2013), eicosanoids (Biringer, 2019) and other lipid groups (Kao et al., 2020), have been associated with AD pathology (Barbash et al., 2017). Other metabolic pathways, such as one-carbon metabolism (Oikonomidi et al., 2016; Troesch et al., 2016; Dayon et al., 2017), glucose (Arnold et al., 2018) and amino acid metabolism (De Leeuw et al., 2017), amongst others (van der Velpen et al., 2019; Xu et al., 2021), are also implicated in AD. Vascular factors are also part of the AD pathology (Bowman et al., 2018; Nation et al., 2019). Yet it is difficult to paint a complete figure of the endophenotypes of AD as (i) there are large inter-individual variations, (ii) most previous studies have focused on single pathway alterations, (iii) biological systems are interdependent and interconnected and (iv) their relevance for the clinical manifestation and disease progression remains unclear. From a clinical point of view, a better understanding of the AD endophenotype is needed not only for a better diagnosis and prediction of clinical disease progression, but also with regard to targeted interventions. To achieve this, systemic biological approaches are required. These approaches should simultaneously consider multiple biological levels, their interactions and their relationships with known features of AD. This is especially relevant as so far, the traditional “one gene, one drug, one disease” hypothesis has mostly failed to produce clinically relevant drugs, likely because of the complex disease pathophysiology of AD (Fang et al., 2020). Drug development hypotheses should therefore take into account the great complexity of AD spanning multiple biological levels, and their clinical relevance.

Such in-depth analysis of a disease phenotype can hardly be performed using only hypothesis-driven targeted approaches that typically focus on one or a few groups of molecules of interest. Applying more comprehensive untargeted, data-driven assessments is better suited to address the extent and complexity of disease-related alterations. The recent development of various omics technologies allows, for instance, to resolve the complexity of the metabolome and proteome (Sancesario and Bernardini, 2018). It is now possible to measure a large number of molecules simultaneously with relatively high throughput resulting in sensitive read-outs of biological status and for exploration of molecular fingerprints of diseases (Jove et al., 2014; Shevchenko et al., 2015; Hasin et al., 2017). As powerful phenotyping technologies, omics significantly accelerate the understanding of mechanisms of pathophysiological alterations that underlie complex diseases such as AD.

However, given that biological systems are interdependent and interconnected, it is necessary to consider multiple single-omics datasets together. These approaches are dubbed multi-omics as they integrate data stemming from multiple biological levels, from genes to transcripts, to proteins and to metabolites. Such approaches afford the opportunity to explore in depth the pathway alterations and molecular mechanisms associated with the disease. In the context of AD, distinct endophenotypes and alterations could be identified and associated with distinct disease manifestations, helping us better understand disease heterogeneity and related clinical relevance. Indeed, it is conceivable that distinct endophenotypes underlie cognitive and non-cognitive clinical manifestations, for example. These approaches also have the potential of identifying altered biofluid molecule profiles that could be used as biomarkers for the diagnosis and prognosis in preclinical or early clinical AD stages. Such biomarkers signatures could also be used for monitoring and following up the effects of clinical interventions on relevant pathologies over time.

## Materials and methods

We conducted a review of the existing literature on multi-omic approaches in Alzheimer’s disease, with a focus on hypothesis-free untargeted approaches that integrated different omics modalities and data types originating from different molecular biology levels in order to capture the dynamic pathomechanisms underlying Alzheimer’s disease. We searched PubMed, MEDLINE and Google Scholar for original articles in English language referenced in these databases between 30th September 2017 and 30th September 2022 using the following search terms: “multi-omics,” “multi omics,” “omics,” “Alzheimer’s disease,” “Alzheimer’s,” “AD” and “neurodegenerative disease.” The titles and abstracts of all references were screened for eligibility. Full texts of potentially eligible references were then retrieved and assessed for inclusion.

To be considered, articles had to include systems biology approaches that considered together at least two different biological modalities at different molecular levels from the following: genomics, transcriptomics, proteomics, lipidomics, metabolomics or ionomics, obtained from human participants with confirmed AD pathology (through validated biomarkers of post-mortem neuropathology analysis). We excluded from our analysis review articles, commentaries or errata, unreviewed pre-prints, articles not using human data or those to which we did not have access.

Relevant information was extracted from the included articles, including omics modalities used, use of an integrative method, as defined according to Ritchie et al. (2015), i.e. the process by which different types of omics data are combined as predictor variables to allow more thorough and comprehensive modelling of complex traits or phenotypes, and their main findings.

## Results

After exclusion of duplicates, we obtained 116 different studies. We excluded 32 review articles, 29 studies that were not multi-omic studies, 13 that did not consider AD patients, 11 that were not performed using human samples or were only *in silico* simulations, 3 unreviewed preprints and 3 to which we did not have access. We therefore considered 33 studies for this review shown in Table 1. Before discussing them, we will address the tools and challenges involved in performing multi-omics studies.

**Table 1:** Multi-omics studies in Alzheimer’s disease considered in this review.

Authors	Omics considered	Biological sample	Clinical features of study cohort	Integrative method?	Findings
Beckmann et al. (2020)	Genomics	Brain tissue	AD	No	*VGF* is causally related to AD and is down regulated in patients.
	Transcriptomics				
Chung et al. (2022)	Genomics	Brain tissue	AD	No	*MGMT* expression and methylation associated with AD pathology and risk.
	Transcriptomics				
Clark et al. (2021)	Proteomics	Cerebrospinal fluid	AD and MCI	Yes	Distinct multi-omics molecular signatures differentially related to AD pathology. Biomarker candidates of AD and cognitive decline.
	Lipidomics				
	Metabolomics				
Cohn et al. (2021)	Proteomics	Brain tissue	AD	No	In late AD tau and neuronal debris are resealed through extracellular vesicles.
	Transcriptomics				
	Lipidomics				
Du et al. (2021)	Genomics	Plasma	AD and MCI	No	Genomic and transcriptomic biomarkers are associated with neuroimaging features
	Proteomics				
Fang et al. (2022)	Genomics	Brain tissue	AD	No	Identification of 103 risk genes for AD. Three drugs associated with decreased risk of AD.
	Transcriptomics				
	Proteomics				
Han et al. (2021)	Genomics	Brain tissue	AD	No	Web-based tool for visualisation of data
	Transcriptomics				
Hu et al. (2020)	Genomics	Brain tissue	General population	Yes	Identification of 16 shared causal pathways between AD and Type 2 Diabetes
	Transcriptomics				
Johnson et al. (2020)	Genomics	Brain tissue, Cerebrospinal fluid	General population	No	Microglial metabolism is associated with both normal ageing and AD and could serve a neuroprotective anti-inflammatory function.
	Proteomics				
Johnson et al. (2022)	Genomics	Brain tissue	General population	No	APOEε4 and cognitive decline are not associated with the same biological pathways.
	Transcriptomics				
	Proteomics				
Lefterov et al. (2019)	Transcriptomics	Brain tissue	AD	No	Differential association of APOE genotype with transcriptomic and lipidomic profiles in AD
	Lipidomics				
Li et al. (2020)	Genomics	Brain tissue	AD and MCI	Yes	Prediction of PET-imaging outcomes improved by multi-omics modelling
	Transcriptomics				
Li et al. (2021)	Genomics	Brain tissue	AD and MCI	Yes	Molecular subtypes are associated with MCI to AD conversion risk
	Transcriptomics				
Madrid et al. (2021)	Genomics	Blood	AD	No	Glypican-2 (GPC2) protein downregulated in AD cases
	Transcriptomics				
Min et al. (2021)	Genomics	Brain tissue	AD	No	Somatic mutations unlikely to be causal for AD
	Transcriptomics				
Nativio et al. (2020)	Transcriptomics	Brain tissue	AD	No	AD affects the epigenome. Disease pathways are affected by chromatin and transcription regulation.
	Proteomics				
	Epigenomics				
Odom et al. (2021)	Genomics	Brain tissue	AD	Yes	Most pathways involved in AD pathology are not picked up by single-omic approaches
	Transcriptomics				
Park et al. (2022)	Genomics	Blood	AD and MCI	Yes	Identification of possible molecular drivers of AD heterogeneity or subtypes
	Transcriptomics				
	Proteomics				

Peña-Bautista et al. (2021)	Genomics	Blood	AD and MCI	No	Lipids and miRNAs involved in fatty acids mechanisms associated with disease.
	Lipidomics				
Rosenthal et al. (2022)	Genomics	Brain tissue	AD	No	Identification of 17 gene clusters of pathways altered in AD
	Transcriptomics				
Ruffini et al. (2020)	Genomics	Brain tissue	AD	No	AD shares many proteomic and transcriptomic pathways with other neurodegenerative diseases
	Transcriptomics				
	Proteomics				
Seyfried et al. (2017)	Genomics	Brain tissue	AD	No	Genetic risk loci for late-onset AD are preferentially enriched in microglia
	Transcriptomics				
	Proteomics				
Shigemizu et al. (2020)	Genomics	Blood	MCI	No	Effective in MCI-to-AD conversion prediction model.
	Transcriptomics				
Song et al. (2021)	Genomics	Blood	AD	No	30 cross-omics blood-based biomarkers associated with AD.
	Transcriptomics				
	Proteomics				
Tasaki et al. (2018)	Genomics	Brain tissue	General population	Yes	Specific set of neuronal genes to controls cognitive decline in older adults
	Transcriptomics				
Tasaki et al. (2019)	Genomics	Brain tissue	General population	No	Cognitive and motor impairment could share an underlying genetic architecture
	Transcriptomics				
	Proteomics				
Wang M. et al. (2021)	Genomics	Brain tissue	AD	Yes	Molecular signatures and gene networks identified for four brain regions. *ATP6V1A is an important driver of AD.*
	Transcriptomics				
Wingo et al. (2022)	Genomics	Brain tissue	General population	No	Many of the neurodegenerative disease causal proteins are shared with psychiatric disorders
	Transcriptomics				
	Proteomics				
Xicota et al. (2019)	Transcriptomics	Plasma	Memory clinic patients	Yes	Potential blood omics signature for prediction of amyloid positivity
	Metabolomics				
	Lipidomics				
Xie et al. (2021)	Genomics	Brain tissue	General population	Yes	Discovery of multi-omic networks associated with brain function and neuroimaging.
	Transcriptomics				
	Proteomics				
Xu et al. (2020)	Proteomics	Plasma	AD and MCI	No	Five lipid modules and 5 protein modules regulating homeostasis and innate immunity are strongly associated with AD.
	Lipidomics				
Yu et al. (2022)	Transcriptomics	Blood, brain tissues	AD	No	Actin cytoskeleton is the most pronounced change in the cerebral cortex and serum of AD patients
	Proteomics				
Zhou et al. (2021)	Genomics	Blood, brain tissues	AD	No	Useful tool for database browsing and network visualisation
	Transcriptomics				
	Proteomics				

AD, dementia of AD type; MCI, mild cognitive impairment.

## Performing multi-omic studies

### Challenges of multi-omic studies

Combining different omics level and data modalities is challenging. Indeed, integrating genomics, transcriptomics, proteomics and other omics data often results in handling very heterogeneous data. This heterogeneity results first from the different analytical performances (e.g. precision, trueness and limit of quantification) that vary from an omics platform to another (even within a given omics field such as proteomics). The obtained quantitative data are also diverse in nature (e.g. counts, intensities, areas under the curve, relative ratios and concentrations) and often transformed and/or treated with different normalisation and data scaling approaches. Another issue is the potential sparsity of data. This is especially true for metabolomics data where multiple molecules can likely be found below the lower limit of quantification, or mass spectrometry-based proteomics when data-dependent acquisition (a data-driven stochastic process of recoding data) is used, generating significant numbers of missing values in datasets. For these reasons, omics datasets must be cleaned and prepared properly before further analysis. In this process, outliers should be treated individually before data integration.

Other challenges include the subsequent statistical analyses. Certain diseases are rarer than others and therefore can cause class-imbalance in datasets. In these cases, weighted approaches or over-sampling should be considered. Additionally, cross-validation or regularisation should be considered. In the context of AD, this could be the case in studies investigating familial AD specifically, as it is estimated less than 5% of AD cases are causes by deterministic genes (Wu et al., 2012). Another challenge specific to the investigation of the endophenotype of AD is the heterogeneity regarding the development of the disease pathology and its clinical presentation. Indeed, AD has a long preclinical and prodromal period, during which distinct pathophysiological processes with their own dynamics might emerge at different times. While multi-omics data are better suited to distinguish differentiate between individual heterogeneity and underlying changes in many participants, this makes direct comparisons between cross-sectional studies difficult.

The major problem facing multi-omics studies however is the famous “curse of dimensionality” whereby much more features (i.e. analytes) are measured compared to the number of datapoints available (Bellman and Kalaba, 1959; Berisha et al., 2021). This means the dimensional space must be reduced by feature selection or extraction approaches such as least absolute shrinkage and selection operator (LASSO) or principal component analysis (PCA) respectively before data integration. In addition, distinct-omics modalities and approaches present individual advantages and challenges. For example, genomic data are unlikely to capture dynamic processes involved in pathophysiological processes. Metabolomics data on the other hand provides a dynamic readout of the disease state but is likely also affected by the environment and nutritional habits.

### Available multi-omics integration tools

From a statistical point of view, there are multiple ways to approach multi-omics data integration (i) concatenation which develops models using a joint data matrix which is formed by combining multiple omics datasets, (ii) model-based approaches which create multiple intermediate models for the different omics data to then build a final model from various intermediate models and (iii) transformation methods which first transform each of the omics datasets into graphs or kernel matrices and then combine of all of them into one before constructing a model. Concatenation approaches, such as deep neural networks (Tang et al., 2019) or Multi-Omics Factor Analysis (Argelaguet et al., 2018), do not require any pre-processing and can select the most discriminating features for a given phenotype but in general do not consider the unique distribution of each data modality. Model-based approaches such as XGBoost (Ma et al., 2020) and PINS+ (Nguyen et al., 2019) facilitate the understanding of interactions amongst different omics for a given phenotype but are not effective if the data are extremely heterogeneous. Finally, transformative methods have the advantage of being able to combine a wide range of omics and portray the relationships between different omics samples. These methods include MOGONET (Wang T. et al., 2021) and NEMO (Rappoport and Shamir, 2019) and are usually more challenging to perform because they are computationally more intensive than other methods. In addition, transforming the data can itself be challenging. Finally, each of these integration types can be supervised (fitting a model with labelled training data and then use it for prediction) or unsupervised (finding the underlying patterns in unlabelled data using input feature variables) which are especially useful for identifying clusters within the data. We refer to Reel et al., for an extensive review of the available machine-learning tools for multi-omics analysis (Reel et al., 2021). Faced with this plethora of available tools, another challenge for researchers is indeed which method to choose. It remains often necessary to practically evaluate a few strategies.

## Multi-omics approaches applied to AD

While multi-omics have been used for over a decade to investigate alterations in other diseases, in particular in the field of oncology (Akhoundova and Rubin, 2022), multi-omic investigations of AD are still in their infancy. Nonetheless, in the past 5 years, several multi-omics studies have been performed on human cohorts and data in the context of AD (Table 1). However, few of these studies are truly integrative. While they consider multiple biological levels, they only investigated these alterations independently of each other. For example, Cohn et al. (2021) analysed proteomics, transcriptomics and lipidomics data derived from microglial extracellular vesicles obtained from cryopreserved brain tissue with neuropathologically confirmed AD. They showed that alteration of microglial function in the late stages of AD leads to the release of neuronal debris, including tau in extracellular vesicles. This suggests that extracellular vesicles could be used as not only a source of future omics data but also as a potential readout of disease state. More recently, also using brain tissue from AD patients, Chung et al. (2022) explored genomics and transcriptomics data and found an association of AD with genetic variants in *MGMT* (O6-methylguanine-DNA methyltransferase, which is involved in DNA damage repair function) amongst women lacking the APOE ε4 allele, the most important genetic risk factor in sporadic (non-familial) AD. Furthermore, the epigenetic regulation of *MGMT* was associated with tau-related pathology. The APOE ε4 allele has also been associated with proteins within the extracellular matrix and glycosaminoglycan-binding proteins in a study considering genomics, proteomics and transcriptomics data obtained from brain tissue derived from the general population (Johnson et al., 2022). Also, considering genomics and transcriptomics obtained from AD confirmed brain tissue, Min et al. (2021) have shown that single-nucleotide variants of AD-candidate genes are not a causal factor of sporadic AD. Non-integrative multi-omics analyses were also applied to investigate alterations in blood-based miRNA levels in relation to genomics data, Shigemizu et al. (2020) revealing that single-nucleotide polymorphism-microRNA pairs can be used to improve the prediction of conversion from mild cognitive impairment (MCI) to AD dementia.

While all these studies provide important and confirmatory clues for the better understanding of the pathophysiological mechanisms of AD and reveal potential new risk-factor and diagnosis tools, they do not fully leverage the power of multi-omics data integration. Firstly, they do not use integration algorithms, as defined according to Ritchie et al. (2015), that consider the interactions that most likely exist between the different biological levels considered. Indeed, no analysis of the combined data was performed but rather each individual data modalities were investigated separately without integrating the different results were integrated into a single framework. Another difficulty is that the most studies only consider two or three biological levels, such as. genomics and transcriptomics, neglecting the downstream events happening at the proteome and metabolome levels. The next most considered modality is proteomics, but very few studies consider lipidomics, metabolomics or ionomics (a term defining the entire elemental composition of a living organism) data, all of which have previously been associated with AD (Zheng et al., 2016; De Leeuw et al., 2017; Gamba et al., 2019; van der Velpen et al., 2019). Of note, it is also possible to consider in multi-omics studies non-molecular data such as neuroimaging and clinical data. For example, investigating both genomics and proteomics of brain tissues derived from MCI and AD patients, Xie et al. (2021) found 20 SNPs from 20 genes associated with the function and structure of six frontal cortex regions. These SNPs were also associated with increased amyloid deposition in these areas. Integration of such clinical and functional data greatly enhances the real-world application chances of the generated results.

Some recent studies have however pursued a true integrative approach. Importantly, Odom et al. (2021) have used such an approach to analyse genomics and transcriptomics derived from brain tissue from AD patients. They have shown that most pathways involved in AD pathology are not picked up by single-omics approaches. Indeed, combining multiple weak signals from a number of individual molecules is more likely to identify key biological hubs or pathway alterations relevant for disease. For example, Wang M. et al. (2021) used post-mortem brain tissue samples with confirmed AD to identify *ATP6V1A* (encoding an ATPase component) as a key network regulator involved in multiple protein networks associated with AD. This suggests that this gene is not only a potential risk factor for AD but could also serve as a target for disease modifications, highlighting the power of integrative approaches for discovering potential disease modifying drug targets.

The common findings of all the studies presented in Table 1 could help build a clearer and more accurate picture of the different processes underlying AD pathophysiology. These muti-omics approaches have provided evidence for alteration of molecular and cellular pathways underlying AD pathophysiology such as the immune system response (including complement activation), haemostasis pathways, energy metabolism and mitochondrial function, lipid homeostasis and processing, extracellular matrix regulation, synapse biology and plasticity, neuronal function, proteostasis, oxidative stress, signal transduction pathways and programmed cell death.

### Exploring pathway alterations related to core aspects of AD pathology

It also necessary to put these results in the context of previous knowledge regarding AD pathology. Whenever possible the identified alterations should be associated with the known core aspects of AD pathology (i.e. amyloid pathology, tau pathology and neurodegeneration). The most obvious route for this is to associate the derived networks or groups of alterations with established biomarkers of AD pathology. One caveat is that such association should be performed *a posteriori* to avoid any selection bias in the considered pathways which would drastically reduce the utility of multi-omics approaches. For example, we have recently investigated in a memory-clinic cohort CSF multi-omics signatures of AD by integrating multi-omics data (Clark et al., 2021). We identified five major dimensions of heterogeneity that together comprehensively explained the endophenotypic variance and were associated with core AD pathology. Exploring these dimensions of variance within the cohort we found that individual CSF biomarkers of amyloid aggregation, neurodegeneration and tau hyperphosphorylation were associated with distinct molecular signatures and pathway alterations. For example, the haemostasis pathway was associated with tau pathology and neurodegeneration, while neuronal function was associated with amyloid pathology.

It is also possible to predict amyloid positivity using multi-omic approaches. Li et al. have proposed a model with high accuracy using genomic and transcriptomic data (Li et al., 2020). While this model outperforms non-multi-omics models, it contains over 200 variables and is therefore not usable in clinical practice. In another study, the authors derived a biomarker signature of early amyloid deposition in asymptomatic individuals (Xicota et al., 2019). This signature contains only five molecules, and it is also derived from omics data obtained from plasma samples. This is especially relevant as blood-based biomarkers are much less invasive and more easily accessible than currently established CSF biomarkers of AD.

Unfortunately, there is currently no consensus or similarity in the biomarker candidates revealed by these studies. Replications of those results are needed before considering any of the biomarker candidates for therapeutic and/or diagnosis use. All the above studies demonstrate that a multitude of pathophysiological process can be linked to the development of AD pathology.

## Molecular alterations with clinical relevance

### Cognitive impairment and cognitive decline

The studies previously mentioned greatly improve our understanding of the underlying mechanisms of AD, but their clinical utility is somewhat limited. Indeed, very few of them provide any useful information about the relationships between specific biological alterations and clinical presentation and prognosis. The already mentioned study by Shigemizu et al. (2020) shows that single-nucleotide polymorphism-microRNA pairs can be used for assessing risk of mild cognitive impairment to AD dementia conversion, but unfortunately the mechanisms driving the conversion are not currently elucidated. Studies by Johnson et al. (2020) and Johnson et al. (2022) have shown that APOE ε4 and the associated extracellular matrix proteins was not associated with cognitive decline whereas mitogen-activated protein kinases (MAPK) were associated with cognitive decline. This suggests that MAPKs are the principal modulators of the cognitive impairment and decline independently of the APOE genotype. According to Li et al. (2021), conversion risk differs between patient groups presenting distinct genomic, proteomic and neuroimaging features. Using proteomics, genomics and transcriptomics data derived from brain tissue, Seyfried et al. identified a genetic risk locus for AD encoding genes involved in microglia and oligodendrocyte function associated with cognitive decline (Seyfried et al., 2017). We have also already mentioned our own studies (Clark et al., 2021), where we identified dimensions of heterogeneity within a cohort associated with AD. Analysing our model deeper, we were able to find molecular signatures predictive of cognitive decline. Both of these signatures contained only four molecules each, taken from multiple biological levels which significantly improved prediction performance of cognitive decline when added to reference models containing only readily available clinical variables.

### Neuropsychiatric symptoms

Besides cognitive impairment, neuropsychiatric symptoms (NPS) are very common clinical features of AD dementia. The presence of NPS alone or in combination with cognitive symptoms form a clinical risk factor for further disease progression (Taragano et al., 2018). The exact underlying pathophysiology of NPS has not been clarified yet. Also, the relationship between AD pathology with the pathological mechanisms of NPS is not clear to date. Especially the question whether NPS independently contribute to cognitive and functional decline needs further clarification. Investigating these open questions through multi-omics approaches seem a reliable option.

Despite the growing number of various omics and multi-omics studies in AD, very few omics studies and, to our best knowledge, no multi-omics approaches currently exist for the investigation of NPS in the elderly. We have previously explored the pathological mechanisms of NPS in cognitively impaired and unimpaired community-dwelling participants in a longitudinal cohort study. Using proteomics data derived from CSF, we found 27 proteins associated with NPS. Enriched pathways analysis revealed different biological pathway alterations related to the single neuropsychiatric symptoms. For example, depression was associated with neuroinflammatory pathways while apathy was associated with cell adhesion and signal transduction pathways (Mroczek et al., 2022). Using the same approach applied to blood-based proteomics data revealed 15 proteins associated with NPS. These proteins additionally were predictive of disease progression, i.e. persistence of NPS over time and associated cognitive decline over 3 years. Interestingly, the associations seemed to be independent of the presence of AD pathology indicating pathways partially distinct from AD (Rabl et al., 2022). Indeed, it appears only 30% of the proteins causative of neurodegenerative disease are implicated in psychiatric disorders (Wingo et al., 2022). These studies underline the need for further omics and multi-omics studies to clarify how NPS alter the clinical course of AD.

## The promise of personalised medicine

There is also a potential for multi-omics approaches to classify AD subtypes by their biological endophenotype in the first place. Such biologically defined subtypes would be devoid of any clinical bias while still allowing for considering the clinical manifestation and disease severity, and progression over time. Defining disease subtypes biologically would also be very helpful for grouping participants in future clinical trials specifically targeting the altered biological pathways. Integration of multi-omics data obtained from cerebral tissue of cerebrospinal fluid will allow to explore and unravel the disease pathomechanisms and could help identify and investigate drug-targets and biological pathways alterations. On the other hand, blood (or systemic)-derived multi-omics data are better suited to identify biomarkers for individual diagnosis, prognosis and monitoring. For example, using readily accessible blood-derived genomics, transcriptomics and proteomics data from patients with cerebral amyloid pathology, Park et al. (2022) have identified possible molecular drivers of AD heterogeneity or AD subtypes. This suggests that specific clinical manifestations of AD could be associated with distinct biological alterations. More generally, the molecular signature of disease could differ from one patient to another, according to cognitive status, age or sex for example (Xu et al., 2021). Therefore, identification of specific molecular signatures is key for (i) better understanding the mechanisms underlying the heterogeneity of AD, (ii) developing specific diagnosis tests based on multi-omics derived biomarkers and (iii) for developing patient-tailored interventions that take into account individual biological specificities. Taken together, such approaches are likely to increase the efficacy of clinical care strategies and interventions.

## Perspectives

**Figure 1:**
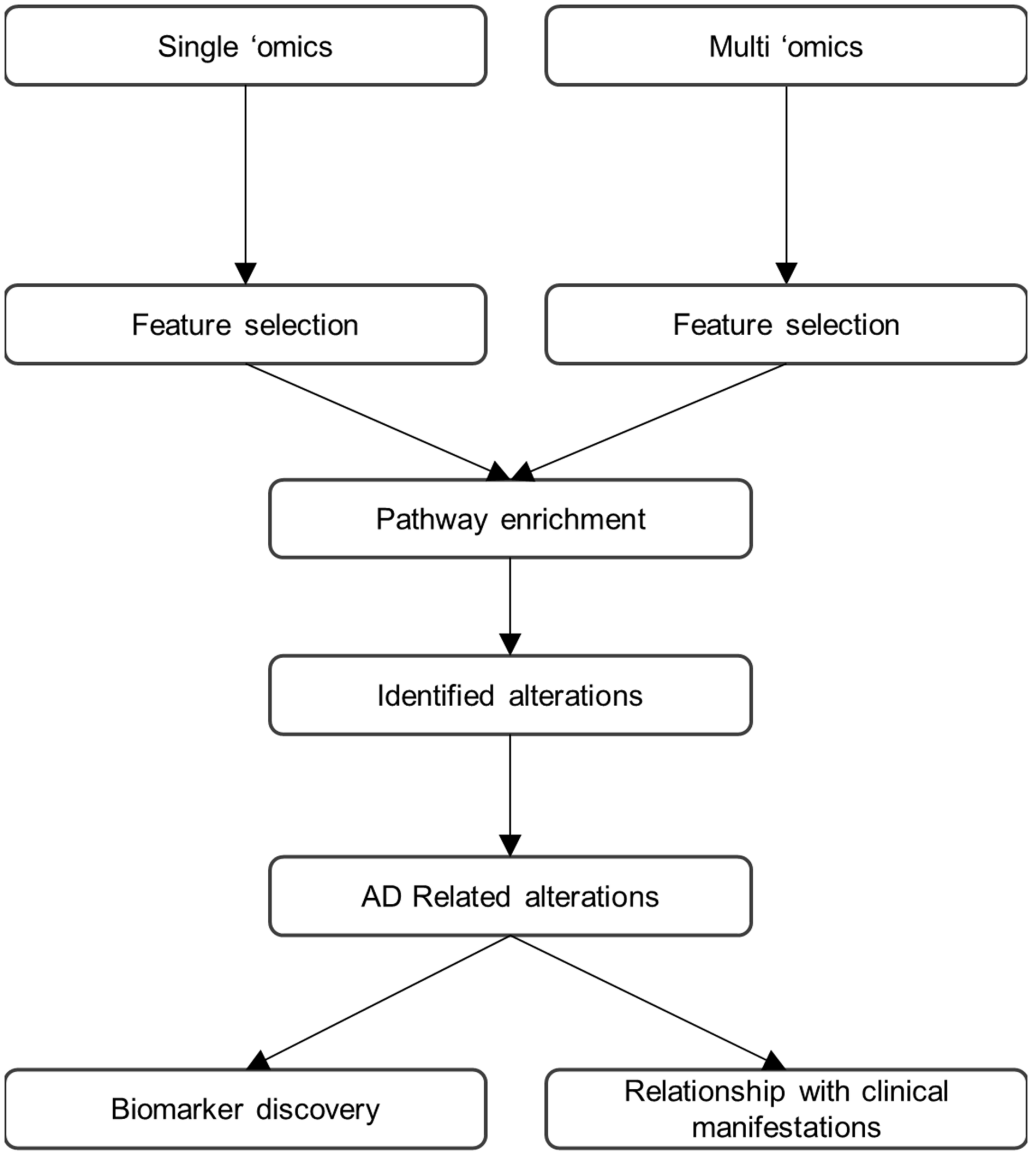
Proposed research framework for future multi-omic studies.

While substantial advance in the understanding of AD have already been made using multi-omics approaches some important limitations remain. First, the findings need to be related to the “core” aspects of AD pathology to better understand their contribution to disease. Indeed, they may be associated with amyloid pathology, tau-related or neurodegeneration, but they could also occur as independent pathophysiological processes. We therefore propose the research framework shown in Figure 1 for multi-omics investigations of AD going forwards. In this paradigm, both untargeted and hypothesis-driven targeted, single omics and multi-omics approaches are performed in parallel and combined to allow for bench-side discovery and for clinical translation (Wood, 2014). Molecules associated with AD should be identified in both single- and multi-omics datasets. The identified molecules should then be subsequently assigned to biochemical pathways using pathway and metabolic network-embedded analysis as well as ontology approaches. Finally, the identified analytes/molecules and pathway alterations should be related to specific aspects of AD pathology at both clinical and molecular levels. Of note, this framework could easily be applied to other neurodegenerative diseases or other pathologies. Moving towards a more unbiased and heterogeneous framework encompassing genes, proteins and molecules contributing to synaptic function, neuroinflammation, transcriptional and translational regulation as well as brain structure and function will be key in unlocking in-depth understanding of AD. It will also allow for the discovery of as-of-yet unimagined biomarkers at various stages of the disease. Such approaches require not only large cohorts but also great amounts of computing power as well as substantial financial resources and therefore can only be undertaken as collaborative multi-centric endeavours (Ritchie et al., 2016).

In addition to performing single- and multi-omics together, the next promising approach could be the meta-analysis of different cohorts as multi-omics data become more and more available in public repositories. While this would drastically strengthen understanding of AD pathology this approach comes with its own challenges. Indeed, there is a vast amount of heterogeneity between the cohorts used and one would also have to deal with data completeness and labelling issues amongst others.

## Conclusion

Despite their relatively small numbers, multi-omics studies in AD have already shed light on the complex evolution of AD pathophysiology, including at individual level. This is especially relevant for a complex disease such as AD where clinical manifestations arise through interactions of multiple biological pathway that result in multifactorial alterations. While many of the identified alterations have been previously investigated in single-omic or hypothesis-driven studies, multi-omics studies have helped heighten our understanding of AD pathophysiology and clinical manifestations and opened perspectives of diagnosis and treatment options beyond the core pathophysiological mechanisms of AD. This will help identify new targets for prevention or treatment for patients at risk of or affected by AD symptoms. In addition, specific alterations detected in peripheral blood plasma or serum could serve as non-invasive and/or more effective biomarker signatures of AD. These have the potential to diagnose AD and its subtypes at early preclinical stages, to predict clinical progression and to monitor the development of relevant aspects of the AD pathology. Indeed, identification of certain metabolic profiles could help clinicians determine whether the patient will experience rapid or rather slow cognitive decline and develop neuropsychiatric symptoms, and therefore help propose better targeted and more efficient clinical care. Importantly, all identified biomarkers or drug targets will need to undergo absolute quantification and validation in independent studies and “real life” cohorts before they can be used in clinical practice.

## Author contributions

CC and JP wrote the manuscript and advised on content. MR and LD provided guidance on content and editing. All authors contributed to the article and approved the submitted version.

## Funding

This work was supported by grants from the Swiss National Research Foundation (SNF 320030_204886) and Synapsis Foundation—Dementia Research Switzerland (grant number 2017-PI01).

## Conflict of interest

The authors declare that the research was conducted in the absence of any commercial or financial relationships that could be construed as a potential conflict of interest.

## Publisher’s note

All claims expressed in this article are solely those of the authors and do not necessarily represent those of their affiliated organizations, or those of the publisher, the editors and the reviewers. Any product that may be evaluated in this article, or claim that may be made by its manufacturer, is not guaranteed or endorsed by the publisher.

## Abbreviations

AD: Alzheimer’s disease
CSF: cerebrospinal fluid
MCI: mild cognitive impairment
NPS: neuropsychiatric symptoms
